# The accuracy and feasibility of respiratory rate measurements in acutely ill adult patients by GPs: a mixed-methods study

**DOI:** 10.3399/BJGPO.2022.0029

**Published:** 2022-10-05

**Authors:** Feike J Loots, Irma Dekker, Ruo Chen Wang, Arthur RH van Zanten, Rogier M Hopstaken, Theo JM Verheij, Paul Giesen, Marleen Smits

**Affiliations:** 1 Julius Center for Health Sciences and Primary Care, University Medical Centre Utrecht, Utrecht University, Utrecht, The Netherlands; 2 Radboud Institute for Health Sciences, Scientific Center for Quality of Healthcare (IQ healthcare), Radboud University Medical Center, Nijmegen, The Netherlands; 3 Gelderse Vallei Hospital, Department of Intensive Care, Willy Brandtlaan, Ede, The Netherlands; 4 Division of Human Nutrition and Health, Wageningen University & Research, Wageningen, The Netherlands; 5 Star-SHL Diagnostic Centres, Etten-Leur, The Netherlands

**Keywords:** respiratory rate, general practice, primary health care, after-hours care, sepsis

## Abstract

**Background:**

Tachypnoea in acutely ill patients can be an early sign of a life-threatening condition such as sepsis. Routine measurement of the respiratory rate by GPs might improve the recognition of sepsis.

**Aim:**

To assess the accuracy and feasibility of respiratory rate measurements by GPs.

**Design & setting:**

Observational cross-sectional mixed-methods study in the setting of out-of-hours (OOH) home visits at three GP cooperatives in The Netherlands.

**Method:**

GPs were observed during the assessment of acutely ill patients, and semi-structured interviews were performed. The GP-assessed respiratory rate was compared with a reference measurement. In the event that the respiratory rate was not counted, GPs were asked to estimate the rate (dichotomised as ≥22 breaths per minute or <22 breaths per minute).

**Results:**

Observations of 130 acutely ill patients were included, and 14 GPs were interviewed. In 33 patients (25%), the GP counted the respiratory rate. A mean difference of 0.27 breaths per minute (95% confidence interval [CI] = -5.7 to 6.3) with the reference measurement was found. At a cut-off point of ≥22 breaths per minute, a sensitivity of 86% (95% CI = 57% to 98%) was found when the GP counted the rate, and a sensitivity of 43% (95% CI = 22% to 66%) when GPs estimated respiratory rates. GPs reported both medical and practical reasons for not routinely measuring the respiratory rate.

**Conclusion:**

GPs are aware of the importance of assessing the respiratory rate of acutely ill adult patients, and counted measurements are accurate. However, in most patients the respiratory rate was not counted, and the rate was often underestimated when estimated.

## How this fits in

Tachypnoea can be the first clinical sign of critical illness such as sepsis. The feasibility and accuracy of the respiratory rate measurement by GPs during assessment of acutely ill adult patients is unknown. This study shows GPs are able to accurately measure the respiratory rate during home visits to acutely ill patients, but in only one of four patient contacts the rate was counted during a period of at least 15 seconds. In the event that the respiratory rate was not counted, tachypnoea was missed in more than half of the patients. Both the feasibility and validity of clinical scores using the respiratory rate as one of the parameters may therefore be limited in primary care.

## Introduction

The respiratory rate is an important sign by which to identify seriously ill patients early in the course of the disease.^1–3^ According to the Airway, Breathing, Circulation, Disability, Exposure (ABCDE) approach, the respiratory rate should be measured as part of the assessment of breathing.^4^ The ABCDE approach is widely used in acute care settings and increasingly taught in GPs' training programmes.^5,6^ The respiratory rate is deemed important for the early recognition of sepsis. In the early stage of sepsis, cytokines and endotoxins increase the respiratory drive, leading to hyperventilation beyond the metabolic needs. In later stages, metabolic acidosis and lung injury induced by sepsis further increase the respiratory drive.^7^ Both the Systemic Inflammatory Response Syndrome (SIRS), and the quick Sequential Organ Failure Assessment (qSOFA) score use the respiratory rate as one of the variables in the score.^8,9^ The cut-off point in the SIRS is >20 breaths per minute, and in the qSOFA it is ≥22 breaths per minute. Also, the respiratory rate is included in warning scores, such as the National Early Warning Score (NEWS) and Modified Early Warning Score (MEWS), which are used to recognise critically ill patients, including patients with sepsis.^10^ Besides for suspected sepsis, the respiratory rate can be used to assess the likelihood and/or severity of acute conditions such as pulmonary embolism, heart failure, pneumonia, and chronic obstructive pulmonary disease (COPD) exacerbations.

Currently, it is not known if GPs can measure the respiratory rate reliably and in which clinical scenarios GPs assess it. This study aimed to examine the accuracy of the respiratory rate measurement by GPs, and evaluate barriers and facilitators for measuring it in acutely ill adult patients.

## Method

The study consisted of observations of respiratory rate measurements during the assessment of acutely ill adult patients. Semi-structured interviews with GPs were held during the same shift in which the GP conducted the home visits. Informed consent was obtained from the participating GPs. The need for informed consent from the patients was waived as all patients received care as usual. Patients were asked if they agreed to the presence of a member of the research team during the GP assessment.

### Setting

The study was conducted between May 2018 and June 2019 at three OOH GP cooperatives located in Ede, Den Bosch, and Uden, serving about 700 000 inhabitants in a mixed urban and rural area. In Dutch OOH GP cooperatives, each patient contact is preceded by telephonic triage by a trained triage nurse supervised by a GP. If possible, telephone advice is given, or patients are asked to come to the OOH GP cooperative for a GP consultation. If this is not feasible, a GP visits the patient at home. If an acute life-threatening condition, such as myocardial infarction, is suspected, an ambulance is deployed to transport the patient directly to a hospital.^11^ At all participating GP cooperatives, contacts with acutely ill adult patients who received a home visit were included in the study to observe respiratory rate measurements. These contacts mainly concerned frail older patients with urgent medical complaints for whom assessment could not be postponed to the next working day. Therefore, all patients were considered acutely ill unless a clear other reason for the home visit was present (for example, determination of death, palliative care, or psychiatric emergencies). At the GP cooperative in Ede only, semi-structured interviews with the GPs were performed.

### Patient observations

During home visits to acutely ill adult patients, a member of the research team (a medical intern) was present during the GP assessment of the patient. GPs were asked to measure the respiratory rate when medically indicated, without specific instruction on which patients should have their respiratory rate measured or how to perform the measurement. During the home visit, the research team member counted the respiratory rate as a reference value by observing the thorax excursions for 60 seconds while the patient was not talking or moving. The reference measurement was usually performed during the physical examination by the GP, but the exact timing and result were concealed for the GP, as the observing researcher was positioned behind the GP and did not provide any information about the measurement to the GP.

Directly after the patient consultation, the GP was asked if they measured the respiratory rate and, if so, what the value and method of measurement were. If the GP counted the respiratory rate for at least 15 seconds, the value was recorded as a counted value. In the event that the GP assessed the respiratory rate without counting the exact rate for at least 15 seconds, it was only recorded whether the estimated rate was ≥22 breaths per minute (cut-off value of the qSOFA). At the GP cooperative where GPs were also interviewed for the study, GPs were asked after each contact with an acutely ill patient to estimate the value (≥22 breaths per minute or <22 breaths per minute), in the event the rate was not counted.

### Qualitative research among GPs

The research team member performed a semi-structured interview with the GP at the beginning of or during the shift. This interview lasted about 10 minutes in total. The interviews were continued until data saturation was achieved. The main topics of the interview were as follows: (1) the frequency and method of the respiratory rate measurement during patient assessments; (2) the clinical scenarios in which they usually measure the respiratory rate; and 3) the perceived relevance of the measurement.

### Data analyses

The quantitative data were analysed using SPSS (version 25). Descriptive analyses were used for the background characteristics of the included patients and GPs. Mean values with standard deviation (SD) were used for normally distributed continuous variables, and median with interquartile range (IQR) for skewed distributions. Pearson’s correlation coefficient assessed the correlation between the counted respiratory rate measurements and reference measurement. A Bland–Altman plot was used to assess systematic differences between the GP and reference measurements. 2×2 contingency tables were calculated at the cut-off value of ≥22 breaths per minute for both the counted and estimated respiratory rate measurements.

The interviews were summarised based on notes taken during the interview, and illustrative quotes were written down in full. Subsequently, the interviews were coded in ATLAS.ti (version 8.2.29.0). The codes were organised based on the topics of the interview.

## Results

The observations of 35 different GPs were included, of whom 14 were also interviewed. Eighteen of 35 (51%) of the GPs were female, and the mean working experience was 16 years. In total, 164 home visits for any medical reason were performed by the 35 GPs. Of these home visits, 130 observations of acutely ill adult patients were included in the analyses (range of 1–7 observations per GP). The excluded home visit contacts concerned determination of death (*n* = 17), child patients (*n* = 3), and patients who were not acutely ill (*n* = 14).

In total, in *n* = 33/130 (25%) of the included patient contacts, the respiratory rate was counted for at least 15 seconds by the GP. At the GP cooperative in Ede, the respiratory rate was counted in *n* = 12/56 (21%) of the patients. Of the remaining 44 patient observations, an estimated respiratory rate (≥22 breaths per minute or <22 breaths per minute) was recorded. At the GP cooperatives in Den Bosch and Uden, in *n* = 21/74 (28%) of the patient contacts, the GP had counted the respiratory rate, and in four cases an estimation was recorded. Table 1 shows the background characteristics of the patients in whom the respiratory rate was counted for at least 15 seconds, compared with the remaining included patients. The median age was 79 years in both groups, with respiratory complaints as the most common reason for the home visit. According to the reference measurement, the mean respiratory rate in the group in which the GP counted the respiratory rate was 21 breaths per minute, compared with 20 breaths per minute in the remaining patients.

**Table 1.** Background characteristics of the included acutely ill patients (*n* = 130), divided by patients in whom the GP did or did not count the respiratory rate for at least 15 seconds

Variable	Respiratory rate counted	
	Yes(*n* = 33)	No(*n* = 97)
Median age, years (IQR)	79 (68–86)	79 (69–85)
Sex, *n* (%)		
Male	15 (45)	50 (52)
Female	18 (55)	47 (48)
Type of complaint, *n* (%)		
Respiratory	12 (36)	16 (16)
Infectious	6 (18)	11 (11)
General malaise	2 (6)	12 (12)
Cardiovascular	4 (12)	9 (9)
Gastrointestinal	4 (12)	14 (14)
Trauma	2 (6)	14 (14)
Neurologic	2 (6)	6 (6)
Other	1 (3)	15 (15)
Urgency at triage, *n* (%)		
U1: immediate response	2 (6)	2 (2)
U2: response as quickly as possible	17 (52)	41 (42)
U3: response within a few hours	13 (39)	53 (55)
U4: response within 24 hours	1 (3)	1 (1)
Mean respiratory rate (SD)	21 (7.3)	20 (6.4)
GP characteristics, *n* (%) (*n* = 35)		
Female sex	14 (42)	43 (44)
>10 years' working experience	25 (76)	74 (76)

IQR= interquartile range. SD = standard deviation.

**Table 2.** Contingency tables of the assessment of the respiratory rate of the GP compared with the reference measurement at a cut-off of 22 breaths per minute (A), GP counted respiratory rate at least 15 seconds, and (B) Estimated respiratory rate by the GP

		Reference measurement		Total
		≥22 per minute	<22 per minute	
A. Counted by GP	≥22 per minute	12	0	12
	<22 per minute	2	19	21
	Total	14	19	33
B. Estimated by GP	≥22 per minute	9	1	10
	<22 per minute	12	26	38
	Total	21	27	48

### Accuracy of the respiratory rate measurement

**Figure 1.**
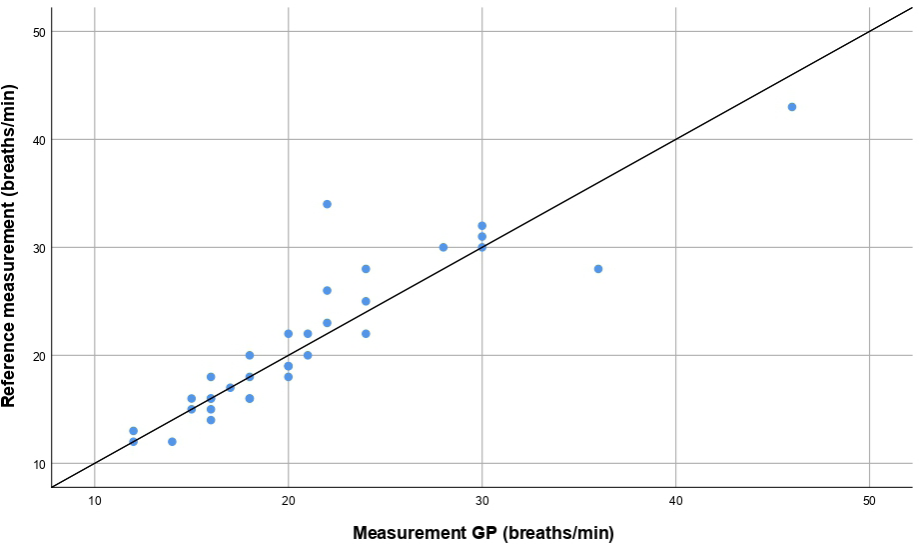
Correlation between the respiratory rate measurements of the GP (counted during at least 15 seconds) and the reference measurement

**Figure 2.**
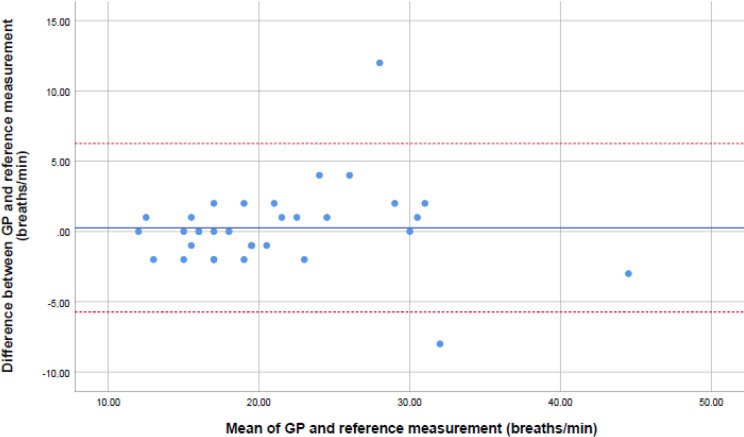
Bland–Altman plot of the differences between the GP measurement of the respiratory rate and the reference measurement

Figure 1 shows the correlation of the counted respiratory rate measurements of the GPs and the reference measurement. The Pearson’s correlation coefficient was 0.91. There was no significant systematic difference between the GP and reference measurement, as shown in the Bland–Altman plot in Figure 2. A mean difference of 0.27 (95% CI = -5.7 to 6.3) breaths per minute was found, and *n* = 28/33 (85%) of the observations were within a margin of error of ≤2 breaths per minute. Contingency tables of both the counted and estimated respiratory rates at a cut-off point of 22 breaths per minute are shown in Table 2. Compared with the reference measurement, the sensitivity for the observation of a respiratory rate ≥22 breaths per minute was 86% (95% CI = 57% to 98%) in patients for whom GPs counted the respiratory rate, and 43% (95% CI = 23% to 66%) in patients for whom GPs estimated the respiratory rate. A specificity of 100% was found (95% CI = 83% to 100%) for the counted observations and 96% (95% CI = 81% to 100%) for the estimated observations.

### Results of the interviews

All 14 interviewed GPs reported assessing the respiratory rate in practice, although some of them reported this as (very) infrequent. None of the GPs performed the measurements routinely in all acutely ill patients. A reported method used to count the respiratory rate was observing thorax excursions for 15–30 seconds, with or without simultaneous palpation of the pulse. Other methods reported by the GPs are lung sound auscultation or palpation of the thorax. Mentioned clinical scenarios to measure the respiratory rate were adult patients with respiratory complaints, suspected infection, and acutely ill children. Other mentioned reasons to measure the respiratory rate were the clinical handover concerning patients referred to the hospital, to objectify shortness of breath, and to complete the overall clinical assessment:

*'You often measure the respiratory rate in really sick patients or patients with dyspnoea*.' (GP8, F, 14yrs experience)

*'It is improbable that the respiration rate influences patient management. In children, on the other hand, I do assess the respiratory rate sometimes.'* (GP7, F, 18yrs experience)

Most interviewed GPs used the respiratory rate with other clinical findings in their final assessment. Reasons not to measure the respiratory rate in all patients can be divided into medical and practical concerns. Most GPs find other vital signs more helpful in their assessment, especially the peripheral oxygen saturation, which is often found sufficient for assessing breathing. Other medical reasons not to measure the respiratory rate are the chance of an abnormal finding without clinical relevance or the feeling it will not change patient management:

*'I combine the respiratory rate with other vital signs and use that to decide how I treat the patient.'* (GP5, M, 38 yrs experience)

*'Since I use a pulse oximeter, I seldom measure the respiratory rate anymore, as this* [the oxygen saturation] *provides me with the information I need.'* (GP6, M, 30 yrs experience)

Practical concerns are the difficulty of counting the respiratory rate in patients who keep talking, are moving, or wear hindering clothes. Also, the needed investment of time was mentioned to play a role in the decision whether to count the respiratory rate:

*'It can be difficult to measure the respiratory rate accurately. You should invest the time for it, and that is not always possible.*' (GP1, F, 12 yrs experience)

## Discussion

### Summary

In the setting of OOH home visits of acutely ill adult patients, GPs counted the respiratory rate during one in four consultations. These counted measurements were found to be accurate. In cases where the respiratory rate was not counted, the rate was often underestimated. A respiratory rate of ≥22 breaths per minute, as used in the qSOFA score, was not noticed in about half of the cases in which the GP did not count the respiratory rate. Based on the GP interviews, respiratory complaints and fever were the most important reasons GPs assess the respiratory rate. The preferred method was to count thorax excursions for 30 seconds (with or without taking the pulse). Reasons not to count the respiratory rate were medical (for example, believed to be less relevant for patient management than other vital signs) and practical (for example, time investment, hindering clothes).

### Strengths and limitations

The most important strength of this study is that GPs were observed during the actual assessment of acutely ill patients in their homes. This study design enabled real-world data to be obtained on the accuracy of the respiratory rate measurement in practice. Another strength is the simultaneous qualitative component of the research, which provided more insight into the feasibility for GPs to count the respiratory rate. Several limitations of the study should also be mentioned. First, the Hawthorne effect may have played an important role during the study. The frequency of measurement of the respiratory rate is probably not representative of the typical situation without the presence of a researcher. As the GPs agreed to participate in a study where the respiratory rate measurement was observed, it is expected that the frequency at which GPs count the respiratory rate is overestimated; the findings should be interpreted as indication of the upper limit of how often (and how accurately) the respiratory rate is measured. Also, the study only focused on respiratory rate measurements during OOH home visits. This may not be representative of other primary care settings. However, all GPs participating in the study also work in office hours, and it is likely assessment of patients in this setting will be done comparably with OOH. Second, results may differ between countries as local GP training programmes probably influence the attitude towards the respiratory rate measurement. Furthermore, a 60-second count of a single researcher as reference measurement was used, which may differ from the true respiratory rate; this is not likely to have influenced the results, however, as differences between the reference measurement and GP counted rates were small. Finally, the number of patient observations was small, leading to wide CIs of the estimated sensitivity of counted and estimated measurements. However, the finding of the low sensitivity for the estimated measurements is robust as the upper limit of the 95% CI is still low at 66%.

### Comparison with existing literature

The authors could not find any previous studies on the accuracy of respiratory rate measurements in general practice. Latten and colleagues, however, assessed the accuracy of the respiratory rate assessment by medical professionals, including GPs, based on video observations.^12^ Overall, 78% of the observations were within a margin of error of 4 breaths per minute, and the accuracy of the GP measurement was comparable with that of other healthcare professionals. The overall misclassification for the qSOFA was 8.9%. In the present study, a somewhat lower misclassification was found in *n* = 2/33 (6%) of the patients for the qSOFA when the GP counted the respiratory rate.

In a study conducted in the emergency department (ED) in The Netherlands, assessments of adult medical patients were observed to assess how the ABCDE approach was applied in practice. The ABCDE approach was used in 26% of the patients. In the event that the ABCDE approach was used, this included measuring the respiratory rate in 92%. Those study results are comparable with the results in the present study, but it should be noted that in the ED, vital signs were already measured during triage before the physician’s assessment. Also, the respiratory rate might be measured by the physician, not as part of the ABCDE assessment.

Several GPs interviewed in the study reasoned that the respiratory rate measurement could be replaced by oxygen saturation. However, increased metabolic need, acidosis, and inflammation are the main drivers of the respiratory rate in sepsis and not decreased oxygenation.^13^ Several questionnaire studies have been performed among clinicians working in the hospital setting, assessing the knowledge of nurses and physicians about pulse oximetry.^14^ The limitations of reading of the peripheral oxygen saturation are poorly understood and 7%–42% of the clinicians believed it provides information about the ventilation of the patient. Measurement of respiratory rate and oxygen saturation are complementary and should not be substituted by one another.^15^

### Implications for research and practice

The finding that GPs in The Netherlands currently measure the respiratory rate only in a minority of the undifferentiated acutely ill patients has several implications. First, the potential signalling function of an increased respiratory rate as an early sign of shock or sepsis is not fully utilised. Second, implementing a sepsis score such as the qSOFA may be difficult, and scores of the qSOFA may not be accurate if the respiratory rate is estimated instead of counted. Education and training of GPs may improve the measurement of the respiratory rate. However, before more extensive efforts are undertaken to encourage respiratory rate measurement by all GPs, it should be proven to be beneficial; as such, more research should be performed in the primary care setting to show the added value of recognising critically ill patients or improving outcomes.

In conclusion, GPs are aware of the importance of assessing the respiratory rate of acutely ill adult patients and can accurately count the frequency. Despite this, the respiratory rate is not counted in most patients, and the rate is often underestimated in these cases, with an important loss of sensitivity to detect a high respiratory rate.

## Data

The dataset relied on in this article is available from the corresponding author on reasonable request.

## References

[1] Fieselmann JF, Hendryx MS, Helms CM, Wakefield DS (1993). Respiratory rate predicts cardiopulmonary arrest for internal medicine inpatients. J Gen Intern Med.

[2] Subbe CP, Davies RG, Williams E (2003). Effect of introducing the Modified Early Warning Score on clinical outcomes, cardio-pulmonary arrests and intensive care utilisation in acute medical admissions. Anaesthesia.

[3] Goldhill DR, McNarry AF, Mandersloot G, McGinley A (2005). A physiologically-based early warning score for ward patients: the association between score and outcome. Anaesthesia.

[4] Thim T, Krarup NHV, Grove EL (2012). Initial assessment and treatment with the Airway, Breathing, Circulation, Disability, Exposure (ABCDE) approach. Int J Gen Med.

[5] Olgers TJ, Dijkstra RS, Drost-de Klerck AM, Ter Maaten JC (2017). The ABCDE primary assessment in the emergency department in medically ill patients: an observational pilot study. Neth J Med.

[6] Mout P, Veld C in ’t, Fraanje WL (2011). Het ABCDE van de acute huisartsgeneeskunde. Huisarts Wet.

[7] Magder S (2009). Bench-to-bedside review: ventilatory abnormalities in sepsis. Crit Care.

[8] Bone RC, Balk RA, Cerra FB (1992). Definitions for sepsis and organ failure and guidelines for the use of innovative therapies in sepsis.The ACCP/SCCM Consensus Conference Committee. American College of Chest Physicians/society of Critical Care Medicine. Chest.

[9] Seymour CW, Liu VX, Iwashyna TJ (2016). Assessment of clinical criteria for sepsis: for the Third International Consensus Definitions for Sepsis and Septic Shock (Sepsis-3). JAMA.

[10] Nannan Panday RS, Minderhoud TC, Alam N, Nanayakkara PWB (2017). Prognostic value of early warning scores in the emergency department (ED) and acute medical unit (AMU): A narrative review. Eur J Intern Med.

[11] Smits M, Rutten M, Keizer E (2017). The development and performance of after-hours primary care in the Netherlands: a narrative review. Ann Intern Med.

[12] Latten GHP, Spek M, Muris JWM (2019). Accuracy and interobserver-agreement of respiratory rate measurements by healthcare professionals, and its effect on the outcomes of clinical prediction/diagnostic rules. PLoS One.

[13] Mauri T, Spinelli E, Pavlovsky B (2021). Respiratory drive in patients with sepsis and septic shock: modulation by high-flow nasal cannula. Anesthesiology.

[14] Elliott M, Tate R, Page K (2006). Do clinicians know how to use pulse oximetry? A literature review and clinical implications. Aust Crit Care.

[15] Cretikos MA, Bellomo R, Hillman K (2008). Respiratory rate: the neglected vital sign. Med J Aust.

